# *Streptococcus agalactiae* Serotype Ia ST7 CC1 in Farmed Nile Tilapia in Latin America: Age-Dependent Disease Expression and Antimicrobial Susceptibility of an Emerging Clonal Lineage

**DOI:** 10.3390/pathogens15050545

**Published:** 2026-05-18

**Authors:** Marco Rozas-Serri, Miguel Fernandez-Alarcon, Mariene Miyoko-Natori, Renata Galetti, Ricardo Harakava, Mateus Cardoso-Guimarães, Ricardo Ildefonso

**Affiliations:** 1 Pathovet Labs, Ribeirão Preto 14025-060, Brazil; 2 Instituto Biológico/IB, São Paulo 04016-035, Brazil

**Keywords:** streptococcosis, *Streptococcus agalactiae*, serotype Ia ST7, tilapia, Latin America

## Abstract

Recently, a strain of *Streptococcus agalactiae* serotype Ia sequence type 7 clonal complex 1 (SaIa ST7 CC1) has emerged in Latin American tilapia aquaculture as an international threat. This study evaluated outbreaks of acute streptococcosis occurring between 2021 and 2025 on commercial Nile tilapia (*Oreochromis niloticus*) farms in six Latin American countries, aiming to integrate molecular, clinical, pathological, and environmental data. In total, 360 moribund or recently dead fish at various production stages (larvae/fry, pre-grow-out, and grow-out) were examined, and 25 *S. agalactiae* isolates were serotyped and subjected to real-time PCR analysis, multilocus sequence typing (MLST), virulence and antimicrobial resistance gene profiling, and antimicrobial susceptibility testing. All isolates belonged to SaIa and shared the same ST7 CC1 MLST profile, forming a highly homogeneous cluster with reference SaIa ST7 CC1 strains previously isolated from tilapia farms in Asia. These results are consistent with the regional spread of a single clonal line. At the larval and fry stages, SaIa ST7 CC1 was associated with hyperacute septicemia, gastrointestinal hemorrhage, and frequent intestinal intussusception, whereas in pre-grow-out and grow-out fish, neurological signs were more prominent, followed by ocular signs, systemic hemorrhages, and coelomic lesions. Histopathological examination showed profuse colonization of the brain, spleen, liver, and intestine by Gram-positive cocci, accompanied by marked acute circulatory and inflammatory lesions and few chronic granulomatous responses, consistent with a rapidly progressing, highly aggressive infectious process. All outbreaks occurred during extended periods of warm water (>32 °C), with large day–night thermal gradients and reduced dissolved oxygen, suggesting that thermal stress may exacerbate disease expression in affected systems. All SaIa ST7 CC1 strains exhibited phenotypic susceptibility to florfenicol and amoxicillin, whereas 84% (21/25) and 100% (25/25) exhibited intermediate susceptibility to oxytetracycline and enrofloxacin, respectively. In total, 5 of the 21 isolates (23.8%) with intermediate susceptibility to oxytetracycline carried tetracycline resistance genes (tetM, tetO). These findings identify SaIa ST7 CC1 as a clinically significant emerging threat associated with thermally facilitated and geographically expanding streptococcosis in tilapia production in Latin America. Immediate priorities include screening imported broodstock using MLST or whole-genome sequencing (WGS), harmonized regional molecular surveillance, climate-adaptive farm management practices, prudent antimicrobial use, and serotype-matched vaccination and breeding strategies that improve both disease and heat resilience.

## 1. Introduction

Global farmed tilapia (*Oreochromis* spp.) production reached approximately 7 million metric tons in 2024, representing an annual increase of about 4–5% relative to 2023 [1] and an estimated market value of around USD 15–15.5 billion, with forecasts projecting growth to USD 21 billion by the year 2035 at a compound annual growth rate close to 3.1%, according to specialized market analyses [2]. Although North America (NAM), Central America (CAM), and South America (SAM) each produce approximately 900,000 metric tons of tilapia annually (about 13% of global production), together they constitute the backbone of the tilapia industry in Latin America (LAM) [3].

This continuous growth is primarily attributable to the species’ high adaptability to diverse environmental conditions and rapid growth, which have enabled its widespread adoption in multiple production systems and climatic zones [4]. Nevertheless, production intensification has occurred without risk-based internal and external biosecurity plans and amid heterogeneous farming practices among LAM-producing countries. This has occurred within the context of increasing environmental variability associated with climate change, which predisposes the sector to the emergence, re-emergence, and/or recurrent outbreaks of bacterial and viral disease with substantial productive, economic, and social impacts.

Over the past two decades, another factor has taken center stage as a key driver redefining host–pathogen–environment interactions for aquaculture and other food production systems: global warming [5,6]. Increasing water temperatures stimulate pathogen replication, virulence, life-cycle dynamics, and disease transmission in fish, while undermining biosecurity measures that were usually developed under less extreme climatic conditions. Tilapia, often characterized as tolerant of environmental variation and even climate change [4,7], are now increasingly exposed to both elevated and fluctuating temperatures, reduced dissolved oxygen (DO) levels, and altered pH and salinity associated with changing precipitation patterns and extreme weather events [8,9,10].

In this context, experimental and field data show that *Streptococcus agalactiae* (group B Streptococcus, GBS), the etiological agent of piscine streptococcosis in tilapia, comprises 10 serotypes (Ia, Ib, II–IX) classified according to capsular polysaccharide antigens (cps) [11], which exhibit marked virulence variation driven by temperature changes [12,13]. In Papua, Negara Bagian Papua (Indonesia), strains that were CAMP-test negative (cfb-deficient) in 2013 became CAMP-test positive by 2023, suggesting adaptive shifts in virulence traits under warmer conditions [14]. Concurrently, temperature-induced decreases in oxygen solubility also result in substantially lower dissolved oxygen concentrations at higher temperatures (e.g., 32 °C vs. 22 °C) and, together with changes to salinity and pH, escalate physiological stress and impair disease resistance [9,10,15,16]. Sustained high water temperatures weaken teleost immunity via oxidative stress and impaired antigen presentation [17], enhance bacterial virulence through upregulated adhesins and toxins [18], and accelerate horizontal gene transfer and the emergence of antimicrobial resistance in aquaculture microbiomes [19].

Over the past five years, the epidemiology of *S. agalactiae* in farmed Nile tilapia across LAM has shifted dynamically [20,21]. Historically, the disease burden has been attributed primarily to *S. agalactiae* serotype Ib (SaIb), including several distinct sequence types (ST 103, ST 260, ST 261, ST 552, ST 553, and ST 927) [22]. However, since 2021, *S. agalactiae* serotype Ia (SaIa), sequence type 7 (ST7), clonal complex 1 (CC1), has spread across several LAM tilapia-producing countries [20,21,23]. Phylogenetic analyses indicate that this lineage originated from a close ancestor of isolates identified in tilapia farms in Asian countries [20]. Comparative studies from Asia have demonstrated that β-hemolytic strains, including SaIa and *S. agalactiae* serotype III (SaIII), are more pathogenic in farmed tilapia than non-hemolytic variants such as SaIb [24,25,26,27,28,29].

Given these circumstances, the objective of this study was to describe and characterize the SaIa ST7 CC1 strain associated with outbreaks of acute streptococcosis in commercially farmed Nile tilapia across six Latin American countries. Specifically, we (i) elucidated the clinical and pathological manifestations of disease across different production stages: larvae/fry (FRY), pre-grow-out (PGO), and grow-out (GO); (ii) identified serotype, sequence type, and phylogenetic relationships among isolates using real-time PCR assays and multilocus sequence typing (MLST); and (iii) profiled key virulence and antimicrobial resistance genes and assessed antimicrobial susceptibility to therapeutics commonly used in regional tilapia aquaculture. This work contributes to the understanding of the molecular epidemiology and pathogenesis of SaIa ST7 CC1 by integrating clinical, pathological, and environmental data and provides a framework for developing climate-adapted surveillance, prevention, and control strategies for streptococcosis in tilapia production systems in Latin America.

## 2. Materials and Methods

### 2.1. Ethical Statement

Pathovet Labs is accredited by the National Council for the Control of Animal Experimentation (CONCEA) of the Ministry of Science, Technology, and Innovation (MCTI) and maintains its own Institutional Committee for the Care and Use of Animals (CEUA), established in accordance with Brazilian federal legislation and CONCEA/MCTI Resolution No. 50/2021. This study was conducted entirely under field conditions on commercial Nile tilapia farms during active streptococcosis outbreaks. Sampling, clinical examination, euthanasia, and transport procedures strictly adhered to institutional standard operating procedures for field disease investigation and diagnostic sampling.

At the time of study design and implementation, the research team did not consider prior ethics committee approval necessary, as this study involved no experimental manipulation beyond routine diagnostic procedures, and all fish were handled under standard commercial farming conditions. These criteria align with those outlined by Bennett et al. [30], who recognized that field collection and diagnostic studies conducted under commercial conditions are frequently exempt from formal Animal Ethics Committee (AEC) review, as they fall outside the scope of procedures typically regulated by such bodies. However, this study was subsequently reviewed by the CEUA-Pathovet Labs Ethics Committee in March 2026, which confirmed that the procedures had been conducted in accordance with the ethical standards applicable to field studies in farmed fish (Protocol No. 004/2026).

Fish were euthanized by immersion in buffered tricaine methanesulfonate (MS-222) at 300–400 mg/L for at least 20 min after cessation of opercular movements, in accordance with the American Veterinary Medical Association (AVMA) Guidelines for the Euthanasia of Animals and institutional animal care protocols. Antimicrobial susceptibility testing was performed in accordance with Clinical and Laboratory Standards Institute (CLSI) guidelines.

### 2.2. Field Fish Sampling

Between 2021 and 2025, acute outbreaks of a presumed bacterial systemic disease occurred in different production stages of commercial Nile tilapia, including FRY (~0.5 to 5 g), PGO (~5 to 80 g), GO (~100 g to harvest), on farms located in six countries (C1–C6) in North America (NAM), Central America (CAM), and South America (SAM) (Table 1). Accordingly, this study included only commercially farmed tilapia, and no animals were maintained under experimental conditions.

All sampled farms operated semi-intensive production systems with variable stocking densities according to production stage: 0.2–2.0 kg/m^3^ in FRY, 3.0–5.0 kg/m^3^ in PGO, and 10–20 kg/m^3^ in GO. Farms in the FRY phase used earthen ponds, fiberglass or geomembrane tanks (PVC/HDPE), or concrete tanks supplied with water from rivers, lakes, reservoirs, or groundwater sources (C1, C3, C5). The PGO and GO phases involved either net-cage systems in rivers, lakes, or reservoirs (C2, C4, C6) or earthen ponds fed by river diversions (C1, C3, C4, C5).

A total of 360 moribund or recently dead specimens (FRY, *n* = 180; PGO/GO, *n* = 180) were intentionally sampled across six countries (C1–C6) (Table 1), with one commercial farm selected per country through the collaborative network. All fish underwent anatomopathological examination, and 92 specimens were selected for histopathological characterization (Table 1). Of the total isolates obtained, 25 were selected for further study due to logistical and budgetary constraints (Table 1): 5 isolates each from C1, C2, C4, and C6; 3 isolates from C3; and 2 isolates from C5.

Water temperature and dissolved oxygen (DO) were recorded twice daily (06:00 h and 14:00 h) throughout the outbreak period using a calibrated multiparameter probe (YSI Pro20, YSI Inc., Yellow Springs, OH, USA). Across all six farms, sustained periods of 3–4 weeks with average water temperatures >32 °C (peaks of 34 °C) and large day–night thermal gradients (ΔT > 5 °C) were recorded during the outbreaks. Nighttime DO concentrations ranged from 1.0 to 1.8 mg/L (20–25% saturation), whereas daytime values ranged from 5.5 to 6.5 mg/L (70–80% saturation). No statistically significant differences were observed among farms or countries for any environmental indicator (one-way ANOVA followed by Tukey’s post hoc test; *p* > 0.05 for all comparisons).

### 2.3. Clinical Signs and Gross Pathology

Moribund and recently dead specimens were selected through intentional sampling from six (06) commercial farms in each participating country. Prior to necropsy, behavioral changes, clinical symptoms, and external macroscopic lesions were recorded for all sampled fish. External examination included systematic evaluation of the skin, scales, fins (including pectoral fin insertion sites), eyes, nasal cavities, oral and oropharyngeal cavities, anus, and gills according to standardized ichthyopathological protocols.

Following surface disinfection with 70% ethanol, fish were systematically necropsied using sterilized instruments. The abdominal cavity of each specimen was opened aseptically to expose the internal organs and allow gross anatomopathological examination. Examined organs included the liver, spleen, kidneys, pericardial cavity, heart, digestive tract, gills, and gonads. The skull was carefully opened to expose the cranial cavity and brain. Finally, tissue samples were collected from multiple target organs selected according to the suspected systemic bacterial pathology and standardized diagnostic guidelines.

### 2.4. Bacteriological Culture and Serotyping

Primary bacteriological procedures were conducted in situ in laboratories available at each farm and consisted of aseptic inoculation of tissue samples from the organs listed in Table 1 onto 5% sheep blood agar (Hardy Diagnostics, Santa Maria, CA, USA), followed by incubation at 28 °C for 24 to 48 h. Colonies suspected to be *S. agalactiae* (e.g., gray-white, flat, and mucoid) were confirmed as Gram-positive cocci or chains of cocci by Gram staining and subsequently subcultured on Brain Heart Infusion (BHI) agar supplemented with 5% defibrinated sheep blood (Heel do Brasil Biomedica Ltd., São José dos Pinhais, PR, Brazil). The same 25 isolates were subsequently used for molecular and phenotypic characterization.

Subsequently, the 25 isolates were serotyped using a commercial latex agglutination kit for GBS types Ia, Ib, and III (SaIII) (ImmuLex™, SSI Diagnostica S/A, Copenhagen, Denmark) according to the manufacturer’s instructions. Briefly, 10 μL of each kit reagent was added to a single GBS colony suspended in 10 μL of saline solution. Reactions were considered positive when agglutination was observed within 30 s. All isolates positive for *Streptococcus* serotype Ia were selected for further analysis.

### 2.5. Histopathological Examination

Tissue samples ~0.5–1 cm^3^ in volume (*n* = 92) were collected from the brain, heart, hepatopancreas, mid-kidney, spleen, stomach, and intestines of each fish. Samples were fixed in 10% neutral buffered formalin (1:10 *v*/*v* sample-to-fixative ratio) for 24 h at room temperature and then transferred to 70% ethanol for long-term preservation. Following fixation, samples were dehydrated through a graded alcohol series and processed according to standard histological protocols. Sections 4 μm thick were cut from each tissue block and stained with hematoxylin and eosin (H&E) and Gram stain for microscopic examination. All areas of each stained organ section were examined using a Leica DM-2000 optical microscope (Leica, Hamburg, Germany) at low (40–100×) and high (400×) magnification. Images were captured using a Leica DFC-295 digital camera (Leica, Hamburg, Germany) and analyzed using the Leica Application Suite Software (LAS X 5.3.1), Image Analysis (Leica, Hamburg, Germany).

### 2.6. Real-Time PCR-Based Serotyping

Genomic DNA from 25 pure bacterial colonies was fragmented with magnetic beads using the L-Beader 24 tissue disruptor (Loccus do Brasil Ltd.a, Cotia, SP, Brazil) according to the manufacturer’s instructions. Bacterial genomic DNA was then semi-automatically extracted using the Maxwell^®^ RSC instrument for the RSC Genomic DNA Kit (Promega Corporation, Madison, WI, USA) according to the manufacturer’s protocol. A volume of 100 µL of supernatant from each sample was suspended in 1 mL of PBS (pH 7.4) in a tube containing 200 µL of lysis buffer and 20 µL of proteinase K.

Specific primers previously described (Supplementary Table S1) were used to identify *S. agalactiae* serotypes Ia, Ib, and III [31]. qPCR was performed in a total reaction volume of 10 μL containing 2X GoTaq qPCR Master Mix (5 μL) (Promega Corporation, Madison, WI, USA), 300 nM of each primer (1.4 μL), and 1.5 μL of DNA template from each sample in duplicate. Reactions were carried out using a QuantStudio 3 Real-Time PCR System (Applied Biosystems, Life Technologies, Carlsbad, CA, USA) under the following conditions: initial denaturation at 95 °C for 2 min, followed by 40 cycles of 95 °C for 15 s and 60 °C for 60 s. Positive controls (DNA of *S. agalactiae* serotypes Ia, Ib, and III), a no-template negative control (UPW), and a negative extraction control were included in every run.

Cycling-threshold (Ct) values were manually set and recorded up to a maximum Ct value of 40, ensuring that thresholds remained constant between runs. Fish samples were considered positive at Ct values <35 and negative at Ct values of 35–40 or when no Ct value was obtained (NoCt), consistent with established analytical criteria for qPCR-based diagnostics in aquatic animal pathogens [32,33]. All qPCR runs included amplification of the Nile tilapia reference gene β-actin as an endogenous extraction control [34].

### 2.7. Multilocus Sequence Typing and Phylogenetic Analysis

Genomic DNA for MLST (multilocus sequence typing) analysis was extracted from pure bacterial colonies, as described above. Fragments ranging from 459 to 519 bp from the reference genes were amplified by PCR using previously described primers [35] (Supplementary Table S1). Amplified products were purified and sequenced bidirectionally using the Sanger method with BigDye 3.1 reagent and a 3500xL capillary sequencer (Applied Biosystems, Life Technologies, Carlsbad, CA, USA). Sequences were aligned using ClustalW and BioEdit software (version 7.7.1) [36]. After alignment of the seven amplicons from each isolate with the *S. agalactiae* PubMLST database (https://pubmlst.org/organisms/streptococcus-agalactiae accessed on 21 September 2025), a sequence type (ST) and a clonal complex (CC) were assigned to each strain.

A phylogenetic analysis was performed using concatenated nucleotide sequences of the seven housekeeping genes obtained through MLST analysis. Evolutionary relationships among 41 *S. agalactiae* isolates (25 LAM ST7 CC1 isolates from the present study and 16 reference strains from Asia and previous LAM isolates available in GenBank) were inferred using the Neighbor-Joining method with Maximum Composite Likelihood distances [37]. Sequences spanning 3455 aligned positions (1st, 2nd, and 3rd codon positions and noncoding sites; gaps removed by complete deletion) were analyzed in MEGA11 version 11 [38]. Nodal support was evaluated using 1000 bootstrap pseudoreplicates, and values ≥70% are shown. Trees were drawn to scale, with branch lengths representing evolutionary distance (substitutions per site). Isolates were classified into serotype groups (SaIa ST7, Salb, SaIII) according to phylogenetic clustering with reference strains. All MLST sequences generated in this study were submitted to GenBank under accession numbers PZ024150 to PZ024324.

### 2.8. Detection of Virulence and Antimicrobial Resistance Genes

Genomic DNA for the detection of virulence genes (VGs) and antimicrobial resistance genes (ARGs) was extracted from pure bacterial colonies, as described above. PCR assays targeting virulence-associated genes, including adhesins, invasins, and immune evasion genes (Supplementary Table S1), were performed using previously described primers and protocols [39,40]. In addition, PCR amplification of antimicrobial resistance genes (ARGs) (Table 2) was performed according to previously described methods [41,42]. Briefly, PCR reactions were prepared according to the published protocols in a total volume of 25 µL per sample using GoTaq^®^ Green Master Mix (12.5 µL) (Promega Corporation, Madison, WI, USA). Amplification was performed in a T100 thermocycler (Bio-Rad Laboratories, Hercules, CA, USA) under the following conditions: initial denaturation at 95 °C for 1 min, followed by 25 cycles of 95 °C for 5 s and 60 °C for 4 min. Amplification products were analyzed by electrophoresis on 1.5% agarose gels stained with ethidium bromide.

### 2.9. Disk Diffusion Susceptibility Testing

Quality control procedures for culture media, biosafety cabinet performance, and antimicrobial disk potency were routinely conducted in accordance with the Clinical and Laboratory Standards Institute (CLSI) guidelines, with adaptations for fish isolates [43]. Isolates were cultured overnight (18–24 h) in a biochemical oxygen demand (BOD) incubator model TE-371/240 L (Tecnal Equipamentos, Piracicaba, SP, Brazil) at 28 ± 2 °C. Fresh overnight cultures were suspended in sterile 0.85% saline and adjusted to a 0.5 McFarland standard. Culture purity was verified prior to each assay; however, longitudinal purity monitoring was not performed, as repeated freeze–thaw cycles may induce mutational events or loss of mobile genetic elements, potentially compromising the genetic integrity of stored isolates [44]. A sterile swab was used to inoculate Mueller–Hinton agar (MHA) plates. Commercial antimicrobial disks corresponding to the agents evaluated in the MIC assay (oxytetracycline, OTC, 30 µg; florfenicol, FFC, 30 µg; amoxicillin, AMX, 10 µg; enrofloxacin, ENR, 5 µg) were aseptically placed on the agar surface. Plates were incubated at 28 ± 2 °C for 18–24 h in ambient air. After incubation, inhibition zone diameters were measured in millimeters [45].

### 2.10. Minimum Inhibitory Concentration (MIC) Determination

Antimicrobial susceptibility testing of *S. agalactiae* fish isolates was performed using the broth microdilution reference method in accordance with CLSI guidelines, with adaptations. MICs were determined according to CLSI M07 procedures [46]. Cation-adjusted Mueller–Hinton broth (CAMHB) was used as the test medium. Antimicrobials tested (OTC, FFC, ENR, AMX) were prepared as stock solutions diluted in sterile 0.85% saline immediately prior to panel preparation. Twofold serial dilutions were prepared in 96-well microdilution plates to cover ten concentrations. The inoculum was standardized to a 0.5 McFarland turbidity standard and diluted to yield a final concentration of 5 × 10^5^ CFU/mL in each well. Plates were incubated at 28 ± 2 °C for 16–20 h in ambient air. MICs were recorded as the lowest antimicrobial concentration showing no visible growth. Quality control (QC) testing was performed in parallel using *Staphylococcus aureus* ATCC 29213, following the QC ranges specified in CLSI M07/M100 and VET01. Interpretive categories (susceptible, intermediate, resistant), when available, were assigned using the most recent CLSI veterinary-specific breakpoints (VET01). When no breakpoints were available for a specific antimicrobial–fish pathogen combination, MICs were reported without categorical interpretation.

## 3. Results

### 3.1. SaIa ST7 CC1 Represents a Single Clone Circulating in Farmed Tilapia Across LAM

**Figure 1:**
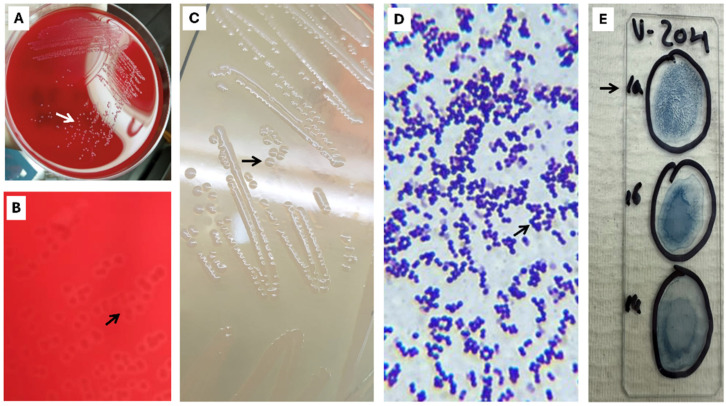
Bacteriological culture and serotyping. (**A**) Inoculation onto 5% sheep blood agar plates in a field laboratory. Bacterial colonies (white arrow); (**B**) Bacterial colonies with a narrow zone of β-hemolytic on 5% sheep blood agar plates (black arrow); (**C**) Cultivation on BHI agar yielded pure bacterial colonies 1–2 μm in diameter, grayish-white in color, smooth, shiny, and translucent (black arrow); (**D**) Gram-positive cocci confirmed from pure colonies (black arrow); (**E**) Agglutination test for *S. agalactiae* serotypes Ia, Ib, and III. Agglutination observed only with serotype Ia (black arrow) indicates that the bacterial suspension contains the Ia capsular polysaccharide and therefore belongs to serotype Ia.

**Figure 2:**
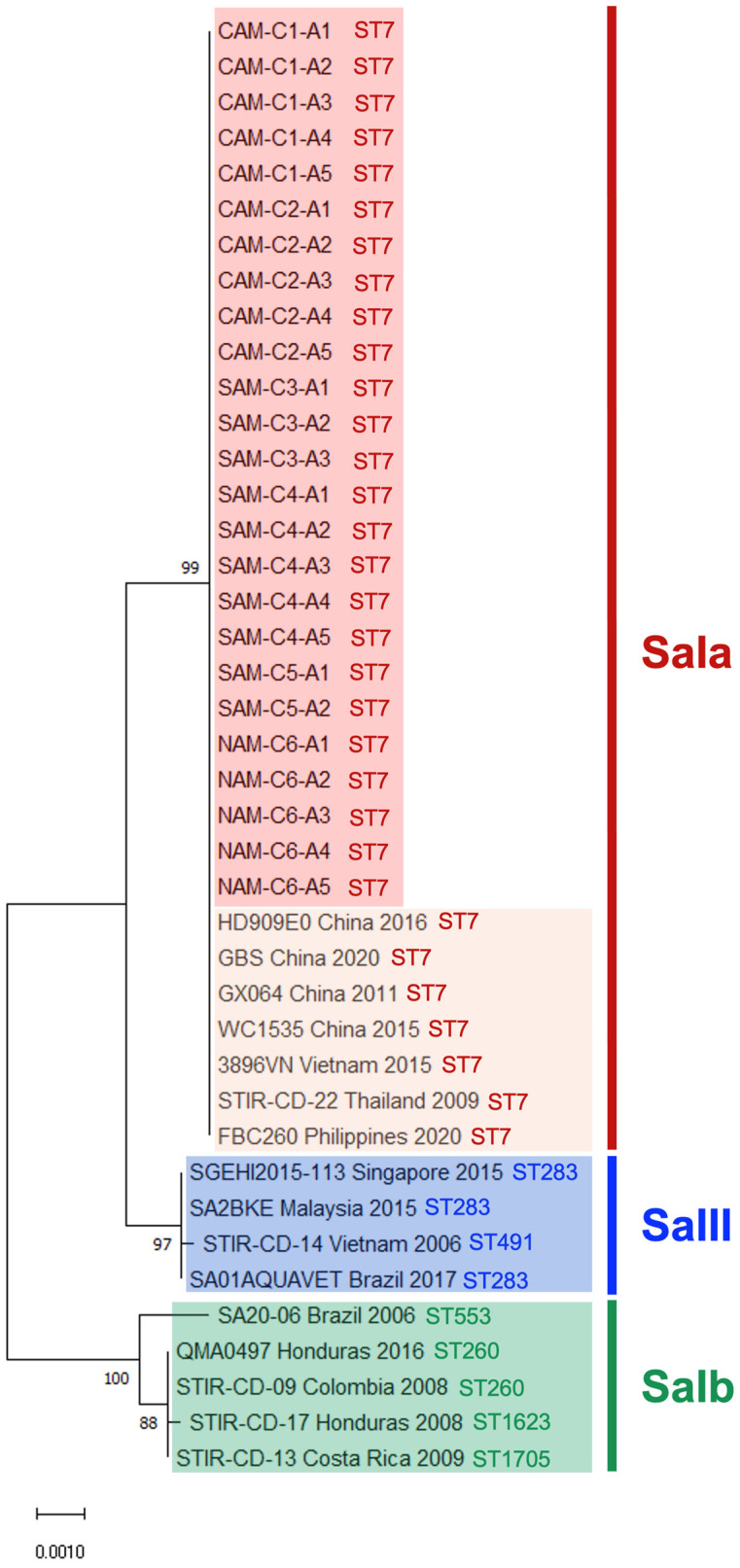
Phylogenetic tree of SaIa ST7 CC1 isolates from LAM and reference strains constructed using concatenated sequences of seven MLST housekeeping genes. Sequences were retrieved from the GenBank and PubMLST databases. The Neighbor-Joining tree of 41 *S. agalactiae* nucleotide sequences shows monophyletic clustering of 25 ST7 CC1 LAM isolates (this study; red box) with Asian and Southeast Asian reference strains (orange box), indicating a single clonal introduction followed by regional dissemination of SaIa ST7. Nine reference isolates of SaIII and SaIb strains from Asia (blue box) and LAM (green box) form distinct basal clades. Numbers at the nodes represent bootstrap support (%) from 1000 replicates; only values ≥ 70% are shown. Scale bar = 0.0010 substitutions per site. The tree was inferred using Maximum Composite Likelihood distances in MEGA11.

All strains obtained from tilapia farms in the six LAM countries were phenotypically characterized as Gram-positive, beta-hemolytic cocci and identified as SaIa by agglutination testing and PCR (Figure 1). Each of the 25 isolates tested positive for SaIa by real-time qPCR (mean Ct = 16.92; SD = 1.44) (Supplementary Table S2). Furthermore, MSLT analysis showed that all isolates shared the same combination of alleles—*adhP*(10), *pheS*(1), *atr*(2), *glnA*(1), *sdhA*(3), *glcK*(2), and *tkt*(2)—characteristic of ST7, which belongs to CC1 (Supplementary Table S2). Phylogenetic analysis further demonstrated that SaIa strains isolated from tilapia farmed in NAM, CAM, and SAM were closely related to each other (99% identity) (Figure 2) and to SaIa strains previously isolated from tilapia farmed in China, Thailand, the Philippines, and Vietnam (99%) (Figure 2). SaIa ST7 CC1 isolates caused similar mortality rates across production stages under field conditions (FRY = 48–52%; PGO/GO = 47–57%) (Table 1). In all outbreaks from which the SaIa ST7 CC1 clone was isolated, water temperatures >32 °C were recorded (Table 1).

### 3.2. Clinical Disease Caused by SaIa ST7 CC1 Is Similar Across LAM but Varies with Fish Age

#### 3.2.1. Clinical Signs and Gross Pathology

**Figure 3:**
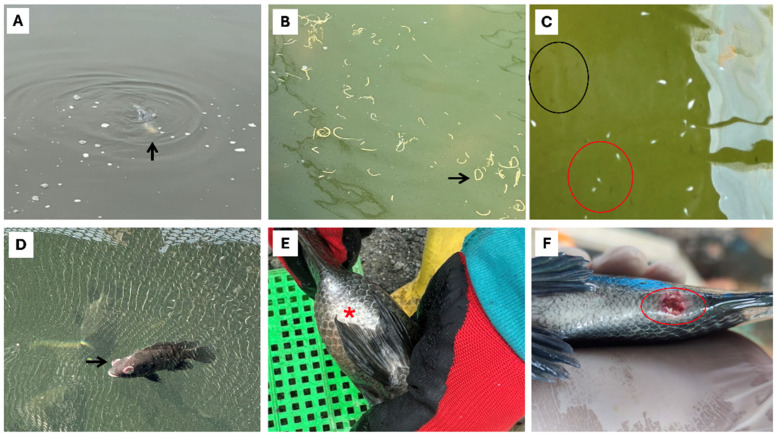
External signs and systemic manifestations in farmed tilapia at different production stages in six LAM countries. (**A**) Erratic swimming, whirling, and lateral or vertical buoyancy in PGO/GO fish (black arrow); (**B**) Multiple fecal strings/mucoid fecal casts floating in the water (black arrow); (**C**) Erratic swimming and turning in fry (red circle) compared to fry with normal swimming behavior (black circle); (**D**) Bilateral exophthalmos (pop-eyes or bulging eyes), corneal opacity (whitish eyes), and hemorrhage (black arrow); (**E**) Skin darkening and abdominal distension (red asterisk); (**F**) hemorrhagic purulent skin ulcers in the perianal region (red circle).

**Figure 4:**
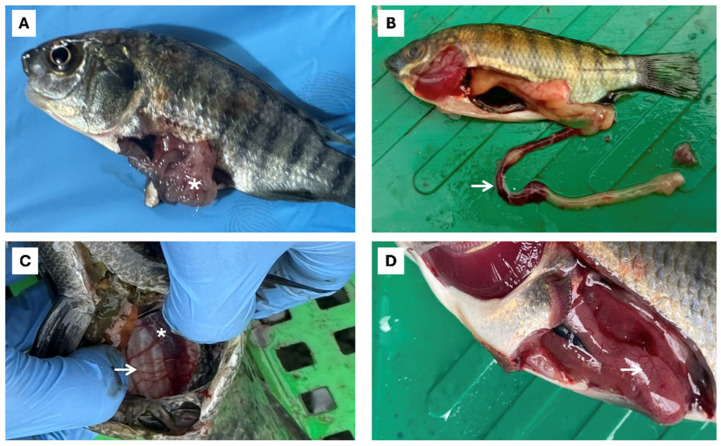
Internal macroscopic lesions in farmed tilapia at different production stages in six LAM countries. (**A**) Thinned intestinal wall with enteritis, serosal hemorrhage, and intestinal intussusception (white asterisk); (**B**) hemorrhagic areas and infarcts associated with intestinal intussusception (white arrow); (**C**) congested swim bladder (white arrow) and renomegaly (white asterisk); (**D**) hepatic hemorrhage and congestion (white arrow).

Outbreaks of piscine streptococcosis caused by *SaIa* ST7 CC1 were reported across all tilapia production stages in LAM (FRY, PGO, and GO) (Figure 3). In larvae/fry, streptococcosis was characterized primarily by anorexia (75.0%), lethargy (83.9%), erratic swimming and whirling behavior (81.7%), cerebral hemorrhage (18.6%), ascites (21.1%), stomach/intestinal hemorrhage (74.4%), intussusception (43.3%) (Figure 4), and, in several cases, hyperacute mortality as the sole clinical manifestation (Table 2). The co-occurrence of cerebral edema and exophthalmos was uncommon in fry (18.3%) but frequent in PGO/GO fish (68.3%).

In larger fish, including both the PGO and GO stages, streptococcosis was characterized primarily by anorexia (86.7%), lethargy (79.4%), erratic swimming and whirling behavior (87.8%), skin darkening (66.1%), unilateral or bilateral exophthalmos (73.9%), and corneal opacity (71.7%). Less frequent findings included fecal strings protruding from the anus and/or floating in the water (25.6%), C-shaped spinal curvature (24.4%), and hemorrhagic purulent skin ulcers in the perianal region (Table 2) (Figure 3).

**Figure 5:**
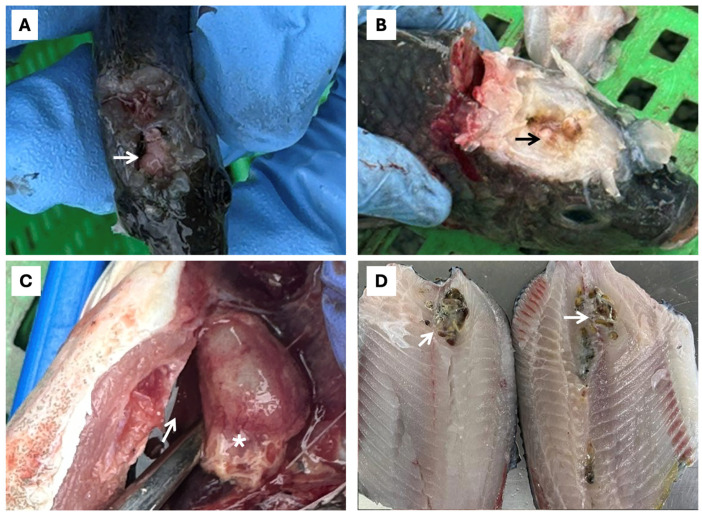
Internal macroscopic lesions in farmed tilapia at different production stages in six LAM countries. (**A**) Meningeal hemorrhage (white arrow); (**B**) thickening and opacity of the meninges and brain, with accumulation of fibrinopurulent exudate in the brain cavity (black arrow); (**C**) thickening and opacity of the epicardium/pericardium, with adherent fibrous deposits (whitish-gray fibrin) (white asterisk) and accumulation of fibrinopurulent exudate in the pericardial cavity (white arrow); (**D**) multiple cavitary foci in skeletal muscle (white arrow), consistent with bacterial abscesses containing bloody, brownish-gray, or yellowish-green fibrinopurulent exudate, melanin, and dystrophic mineralization.

Abdominal distension associated with gas accumulation in the swim bladder or hemorrhagic fluid in the coelomic cavity (68.3%), pericarditis or whitish discoloration of the heart (62.2%), and the presence of yellowish purulent material and meningeal opacity (62.8%) (Figure 5) were also observed (Table 2). Additional notable findings included irregular hepatic appearance and coloration, with mixed pale and congested areas (68.3%) (Figure 4), and abscesses containing purulent material and melanosis in skeletal muscle from fish weighing 100 g up to harvest weight (15.6%) (Figure 5).

#### 3.2.2. Histopathology

**Figure 6:**
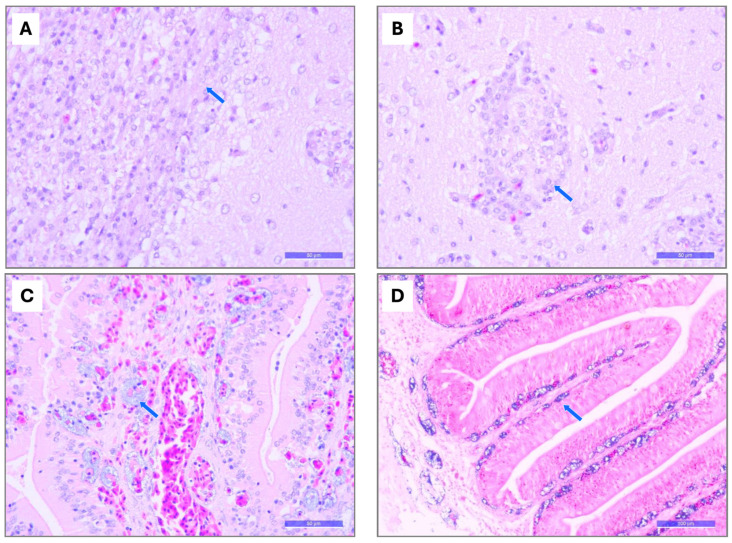
Main microscopic changes in tissues from farmed tilapia at different production stages in six LAM countries. (**A**) Brain (H&E): thickening of the meninges with mononuclear inflammatory cell infiltration (blue arrow) (bar = 50 um). (**B**) Brain (H&E): histiocytic perivascular cuffing (blue arrow) (bar = 50 um). (**C**) Intestine (H&E): hyperemia and the presence of coccoid bacteria in the vascular lumen of the chorion (blue arrow) (bar = 50 um). (**D**) Intestine (Gram): Gram-positive cocci in the submucosa and lamina propria of the villi (blue arrow) (bar = 100 um).

**Figure 7:**
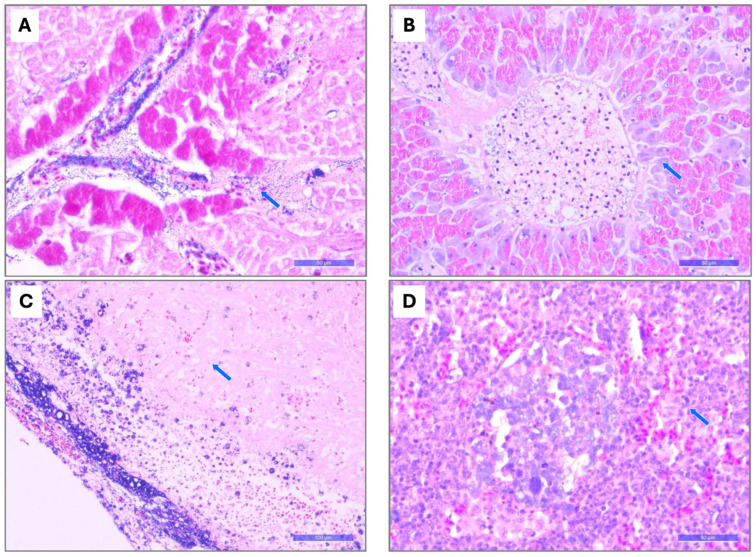
Main microscopic changes in tissues from farmed tilapia at different production stages in six LAM countries. (**A**) Liver (Gram): presence of Gram-positive cocci within vascular lumina and perivascular spaces of the hepatopancreas (blue arrow) (bar = 50 um). (**B**) Liver (H&E): vacuolar degeneration in the hepatic parenchyma with the presence of coccoid bacteria within the vascular lumen (blue arrow) (bar = 50 um). (**C**) Heart (Gram): thickening of the epicardium with numerous Gram-positive cocci (blue arrow) (bar = 100 um). (**D**) Spleen (H&E): severe diffuse splenic capillary dilation containing coccoid bacteria (blue arrow) (bar = 50 um).

Histopathological examination of infected fish revealed marked microscopic changes (Supplementary Figure S1), including intestinal mucosal hyperemia containing numerous coccoid bacteria, accompanied by mild-to-severe hemorrhage and a mild to-moderate mixed inflammatory reaction (89.3%); gastric mucosal hyperemia containing numerous coccoid bacteria (Figure 6), with epithelial ulceration and a mild mixed inflammatory reaction (67.9%); acute diffuse mixed meningitis with moderate multifocal necrotizing mixed encephalitis (42.9%) (Figure 6); and severe diffuse splenic capillary dilation containing coccoid bacteria (Figure 7), together with hyperplasia of melanomacrophage centers (42.9%). Consistent with the gross hepatic lesions observed in larger fish, microscopic hepatic changes were characterized by intravascular and perivascular coccoid bacteria within the hepatic parenchyma, accompanied by acute perivascular histiocytic inflammation and hyperemia (37.0%) (Figure 7), moderate bile duct hyperplasia within the hepatic parenchyma (21.4%), and moderate centrilobular vacuolar hepatopathy (10.7%).

Overall, most fish showed extensive colonization by Gram-positive cocci (Figure 6 and Figure 7), particularly within the bloodstream and multiple tissues and organs, including the stomach, intestines, brain, heart, spleen, and liver, confirming the systemic nature of the infection and suggesting hematogenous bacterial dissemination. Granulomatous lesions, which are commonly associated with chronic streptococcal infection, were infrequently observed, suggesting a hyperacute course of infection. Although inflammatory cell infiltration was generally minimal, phagocytic cells containing bacteria were observed in the spleen and meninges/brain.

### 3.3. LAM SaIa ST7 CC1 Shows a Uniform Virulence Profile

Although LAM SaIa ST7 CC1 isolates showed remarkable uniformity in the virulence genes (VGs) analyzed, minor differences allowed classification into two profiles, arbitrarily designated A and B in this study (Table 3). The predominant VG profile was *spb1-bca-cfb-bac-dtIR* (profile A) (Supplementary Figure S2), which was present in 60% (15/25) of isolates, followed by the *spb1-bca-cfb-dtIR* (profile B) profile, identified in 40% (10/25) of isolates. All isolates from C1, C3, C4, and C5 exhibited profile A (4 out of 6 countries), whereas isolates from C2 and C6 exhibited only profile B. None of the isolates from any country carried *sodA* or *scpB*. Supplementary Table S3 systematically compares the virulence profiles identified in this study with those of SaIa isolates previously described in China, Thailand/Vietnam, Indonesia, and Egypt.

### 3.4. LAM SaIa ST7 CC1 Exhibits a Similar Pattern of Antimicrobial Susceptibility

None of the analyzed SaIa ST7 CC1 isolates showed antibiotic resistance genes (ARGs) associated with erythromycin, phenicols, or lincomycin resistance (*ermB*, *ermTR*, *mefA*, *linB*, *fexA*, *fexB*) (data are shown in Table 4). However, 20% (5/25) of isolates recovered from fish in three of the six countries carried ARGs associated with OTC resistance (*tetM* or *tetO*) (Table 4; Supplementary Figure S3). Phenotypically, isolates exhibited either susceptibility (16%, 4/25) or intermediate susceptibility (84%, 21/25) to OTC (Table 4). Among isolates with intermediate susceptibility, 23.8% (4/21) carried *tetM* or *tetO* (Table 4). The four susceptible isolates showed an MIC of 0.5 µm/mL for OTC, whereas the 21 isolates with intermediate susceptibility (I) showed MIC values ranging from 1 to 4 µm/mL (Table 4).

No SaIa isolate carried ARGs associated with FFC resistance (*fexA*, *fexB*), and all isolates were classified as susceptible based on inhibition zones ranging from 30 to 36 mm (Table 4). Among these isolates, 84% (21/25) showed MIC values < 1 µm/mL, whereas 16% (4/25) showed MIC values of 2 µm/mL for FFC. Similarly, all isolates (25/25) showed susceptibility to AMX, with inhibition zones ranging from 30 to 46 mm and MIC values ≤ 0.5 µm/mL (Table 4). Finally, all isolates (25/25) showed intermediate sensitivity to ENR, with inhibition zones ranging from 21 to 26 mm and MIC values of 4 µm/mL (Table 4). Overall, the phenotypic susceptibility data suggest that the ST7 CC1 clone currently circulating in LAM has not yet acquired clinically relevant antimicrobial resistance, although continued surveillance remains warranted.

## 4. Discussion

### 4.1. Molecular Epidemiology

The high genetic homogeneity observed among SaIa ST7 CC1 isolates reflects the recent clonal expansion occurring in LAM tilapia production from a molecular epidemiological perspective [20,25,40,47]. Although MLST is useful for initial molecular epidemiological investigations, it provides limited resolution for reconstructing introduction routes, microevolutionary dynamics, or the number of independent dissemination events; therefore, whole-genome sequencing (WGS) is strongly recommended for future studies. The close phylogenetic relationship between these new isolates and Asian SaIa ST7 CC1 strains suggests an epidemiological connection between the two regions, consistent with the historical movement of live tilapia broodstock and fingerlings [48]. Although the available data are insufficient to reconstruct direct transmission pathways, introduction through trade in live fish remains a plausible scenario that should be investigated in import risk analyses.

### 4.2. Pathological and Clinical Disease

Clinically, SaIa ST7 CC1 produced a consistent but stage-dependent disease pattern across all farms assessed [49]. In FRY, hyperacute septicemia characterized by anorexia, lethargy, erratic swimming behavior (swimming in circles), severe gastrointestinal hemorrhage, and frequent intestinal intussusception predominated and rapidly progressed to high cumulative mortality within short time periods [50]. This rapid systemic dissemination often resulted in death within 48 h, preventing the development of distinctive neurological signs or generalized hemorrhage [51,52,53,54,55]. Although intestinal intussusception is rarely documented in piscine streptococcosis, it has been reported in parasitic, viral, and bacterial enteritis [56]. The detection of ST7 in larvae weighing as little as 0.5 g is consistent with either early horizontal transmission in hatcheries or vertical transmission, although this study did not directly investigate parental infection. Vertical transmission of *S. agalactiae* from broodstock to fry has been demonstrated previously in tilapia, including colonization of the gonads of asymptomatic breeders [52,53,54,57].

Neurological and ocular signs, including erratic swimming, loss of buoyancy control, exophthalmos, and corneal opacity, commonly occurred alongside generalized hemorrhagic lesions and coelomic changes in PGO and GO fish [40,51,52,53,54,55,58]. The absence of exophthalmos in fry despite cerebral involvement likely reflects the compliance and incomplete ossification of the skull, which may accommodate increased intracranial pressure without retrobulbar extension [59], together with a hyperacute septicemic course that precedes sustained edema formation [60], an immature BBB, an undeveloped retrobulbar space, and a less organized inflammatory response than adult fish [53].

In adult fish, externally expelled undigested fecal material (25.6%) was identified as a clinically relevant manifestation of streptococcosis in tilapia. Although not pathognomonic, similar fecal strings have been reported in 20–40% of experimentally infected fish [61]. In addition, progressive skeletal muscle abscesses with dystrophic mineralization and cavitation (15.6%), particularly in the caudal peduncle region, may substantially impair fillet quality, increase carcass condemnation, and potentially elevate zoonotic risk [24,39,62].

The occurrence of outbreaks across all production phases, from larvae to harvest-size fish, underscores the pathogen’s capacity to evade host defenses regardless of age-related immunocompetence [53]. Recent outbreaks involving strains such as ST283 have likewise been documented in fish of all sizes, including fry, harvest-size adults, and broodstock, confirming the capacity of these lineages to affect multiple life stages and potentially cross species barriers as zoonotic pathogens [29,49,63,64].

Histopathological analyses revealed extensive colonization of the brain, spleen, liver, and intestine by Gram-positive cocci, accompanied by severe acute circulatory and inflammatory lesions in the affected organs and an absence of substantial chronic granulomatous responses. The rapid systemic dissemination, marked neurotropism, and minimal chronic inflammation are indicative of a highly virulent hyperacute pathogenesis associated with *S. agalactiae* lineages infecting sensitive tilapia hosts. Comparative pathogenicity studies have demonstrated that ST7 consistently induces more severe histopathological lesions in the brain, liver, and spleen than ST283, suggesting differences in tissue tropism and pathogenic mechanisms [40]. The microscopic lesions observed in the present study are consistent with previous descriptions of SaIa infection and further demonstrate the capacity of SaIa ST7 CC1 to cross the blood–brain barrier (BBB) and establish severe meningoencephalitis in juvenile and adult farmed tilapia.

Although the precise route of central nervous system (CNS) invasion was not directly investigated in the present field study, several well-characterized mechanisms by which GBS traverses the BBB are likely relevant to the neurological pathology observed. These mechanisms include transcellular invasion of brain microvascular endothelial cells (BMECs) mediated by the surface adhesins SfbA [65], Srr-1 [66], and lipoteichoic acid [67]; paracellular disruption of tight junction integrity driven by β-hemolysin/cytolysin [68] and hyaluronidase [69]; and exploitation of the host plasminogen system to acquire surface-associated proteolytic activity that facilitates extracellular matrix degradation [70]. More recently, direct BMEC lysis by GBS was demonstrated in zebrafish models, representing an additional mechanism of CNS invasion [71]. Collectively, these mechanisms are consistent with the rapid progression, high bacteremia, and acute inflammatory lesions characteristic of SaIa ST7 CC1 infection documented in the present study. Experimental studies directly targeting these virulence pathways in tilapia models are warranted to clarify their relative contributions to CNS invasion.

The repeated temporal association between SaIa ST7 CC1 outbreaks and prolonged periods of elevated water temperature indicates that thermal stress may be a key factor amplifying disease expression within affected production systems [48,72]. All affected farms experienced sustained water temperatures exceeding 32 °C, pronounced day–night thermal fluctuations, and low dissolved oxygen concentrations, conditions known to impair physiological resilience and immune performance in tilapia. Although comparable environmental data from unaffected farms were unavailable for relative risk assessment, the temporal concurrence between outbreaks and thermal anomalies, combined with existing experimental evidence, supports a model in which elevated temperature and associated stressors reduce the threshold for SaIa ST7 CC1 disease expression.

Genetically improved lines, including the widely disseminated GIFT strain and related commercial derivatives, were selectively bred for rapid growth [73,74,75,76,77] and feed efficiency [78,79] under optimal aquaculture temperatures (27–30 °C) [80,81,82]. However, genetically improved farmed tilapia exhibit significantly lower heat tolerance than native or locally adapted Nile tilapia strains, with differences in lethal thermal thresholds ranging from 3.6 °C to 4.0 °C [77,83,84,85]. Concurrent evidence indicates that heat stress compromises tilapia immune function [86,87] while facilitating the expression of bacterial virulence factors [13,77,87].

At the same time, biosecurity protocols designed for relatively stable thermal conditions may become inadequate when production systems are exposed to temperatures > 32 °C [6,14,88,89]. The 2023–2024 El Niño phenomenon generated significant environmental disturbances across, particularly water scarcity and elevated temperatures. Although development and validation of a predictive risk model were beyond the scope of the present study, the findings identify several key variables, including the movement of unscreened animals and the interaction among elevated temperature, low dissolved oxygen, fish age, and clonal lineage, that should be incorporated into future targeted risk-modeling studies and climate-adaptive farm management strategies aimed at reducing-associated disease spillover.

### 4.3. Virulence Gene Architecture and Host-Adapted Pathogenesis

The highly conserved VG repertoire in SaIa ST7 CC1 isolates recovered from diverse environments across six LAM countries suggests that the clone has established a stable set of determinants that confers a substantial selective advantage in tilapia, regardless of local environmental conditions [39,90]. In contrast, ST7 isolates from non-tilapia hosts are more heterogeneous, suggesting that adaptation to tilapia has been mediated by host-specific genomic specialization [39,90,91].

The predominant VG profile detected in LAM isolates (*spb1-bca-cfb-dltR*), defined by the universal absence of *sodA* and *scpB*, differed from the ten profiles identified in seminal Chinese tilapia isolates, in which the *dltR-bca-sodA-spib-cfb-bac* combination was common [39]. This regional divergence highlights the genomic plasticity of *S. agalactiae* and suggests a model of local adaptation involving both the acquisition and loss of specific determinants [22,26,39,92].

The conserved presence of *cfb* (CAMP factor) in all isolates in this study, despite its variable frequency in human and bovine SaIa ST7 isolates [39,40,93,94,95], suggests that CAMP-mediated cytotoxicity is a critical determinant of pathogenesis in tilapia, in which phagocyte lysis represents a central immune-evasion mechanism. *S. agalactiae* strains that were CAMP-test negative (lacking the *cfb* gene) in 2013 were reported as CAMP-test positive by 2023, suggesting adaptive shifts in virulence traits under warmer conditions [14]. In contrast, the pervasive absence of *sodA* and *scpB* suggests that these strains have shifted from a paradigm based on oxidative stress resistance and complement inactivation to one that is more rapid and cytolytic in nature, mediated by CAMP factor and capsular C antigen (*bca*), supplemented with adhesion/invasion (*spb1*), resistance to antimicrobial peptide killing (*dltR*), and evasion of innate immunity (*bac*).

This profile is mechanistically consistent with the observed pattern of rapid bacteremia, hemorrhagic septicemia, and meningoencephalitis. Additional virulence factors, including *cylE*, *lmb*, and *hylB*, which are common in other piscine CC1 lineages [26,40,47,96], were not investigated in this study but should be prioritized in future genomic analyses to fully characterize the neurotropic phenotype. These findings have immediate implications for vaccine design [20,23], supporting strategies that target conserved SaIa ST7 CC1 antigens and suggesting that prime-boost regimens, rather than single injectable doses, may be required to elicit effective protection.

### 4.4. Antimicrobial Susceptibility and Early Resistance Alarms

All SaIa ST7 CC1 isolates were phenotypically susceptible to FFC and AMX according to the interpretive criteria applied, suggesting that this emergent clone has not yet developed robust resistance to key aquaculture therapeutics used in LAM [97]. However, 84% and 100% of the isolates showed intermediate susceptibility to OTC and enrofloxacin (ENR), respectively. This pattern contrasts with reports from other geographic regions, where *S. agalactiae* and related fish-pathogenic streptococci exhibit reduced susceptibility or established resistance to commonly used antimicrobials [47,98,99].

The intermediate susceptibility to OTC may be preliminarily associated with the sporadic detection of *tetM* and *tetO* genes in ST7 CC1 isolates. However, since this study assessed only the presence or absence of these genes on mobile genetic elements, without evaluating their functional expression, the clinical and epidemiological significance of this finding remains uncertain. More detailed genomic analyses, including WGS to elucidate the genetic context of ARGs, together with functional studies examining gene expression under different antimicrobial and environmental conditions, will be necessary to characterize the trajectory and stability of resistance in this clone.

### 4.5. Implications for Surveillance, Biosecurity, and Future Research

Integrated evidence shows that SaIa ST7 CC1 constitutes an emerging threat to tilapia aquaculture in LAM, with high virulence across production stages and associations with geographic dissemination, thermal stress, and the early acquisition of ARGs. Effective control will require integrated interventions across multiple scales rather than farm-level solutions alone. Priority measures include implementing molecular screening (MLST or WGS) of imported broodstock and fingerlings to prevent the introduction and re-introduction of high-risk clones; establishing harmonized regional outbreak-reporting and molecular-surveillance networks to monitor SaIa ST7 CC1 and related lineages; and incorporating climate-adaptive management strategies, such as temperature and dissolved oxygen monitoring, stocking-density adjustments, and movement restrictions during periods of elevated thermal risk. The development and deployment of serotype-matched vaccines targeting SaIa ST7 CC1, within the framework of broader biosecurity programs, will ultimately be essential to reduce antimicrobial dependence and increase the resilience of tilapia production systems.

Future studies should include WGS of SaIa ST7 CC1 isolates from multiple countries and time points to investigate virulence determinants, antimicrobial resistance gene content, and microevolutionary patterns under regional selective pressures. Controlled infection experiments in tilapia with distinct genetic backgrounds under standardized temperature and environmental conditions would help clarify the relative contributions of pathogen genotype, host lineage, and thermal stress to clinical disease outcomes and survival. These efforts, combined with longitudinal field studies integrating environmental monitoring, production data, and pathogen genomics, will provide the basis for improving risk models, informing selective breeding programs for disease and heat tolerance, and guiding evidence-based policies on antimicrobial use and fish movement within the LAM tilapia industry.

## 5. Conclusions

The cluster of strains identified as SaIa ST7 CC1, associated with acute streptococcosis outbreaks in Nile tilapia across six countries in LAM, reflects the recent emergence of a clonal lineage exhibiting uniform yet age-dependent clinical and pathological disease expression. In larvae and fingerlings, hyperacute septicemia with severe gastrointestinal involvement predominates, whereas in pre-grow-out (PGO) and grow-out (GO) fish, a systemic neurotropic disease with significant ocular and coelomic lesions predominates.

All field outbreaks coincided with intervals of elevated water temperature exceeding 32 °C and low dissolved oxygen, reinforcing the role of thermal and environmental stress as key amplifiers of disease expression in intensively farmed tilapia under anthropogenic climate warming. Although SaIa ST7 CC1 retains phenotypic susceptibility to critical aquaculture antimicrobials, the recent detection of *tetM* and *tetO* in a subpopulation of isolates suggests that tetracycline resistance determinants are already present and may emerge under inappropriate antimicrobial use.

These findings establish SaIa ST7 CC1 as a regionally expanding threat to Latin American tilapia aquaculture, requiring coordinated action, including molecular screening of imported broodstock and fry, harmonized surveillance networks, climate-adaptive farm management, serotype-matched vaccination, and antimicrobial stewardship. Collectively, these measures aim to reduce production losses and strengthen aquaculture resilience against transboundary pathogens under accelerating climate change.

## Figures and Tables

**Table 1:** Summary of *S. agalactiae* isolates recovered from acute outbreaks of a systemic disease in Nile tilapia of different age ranges on different commercial farms in six countries of LAM. Average cumulative mortality rates on each farm during the outbreaks and recorded water temperature ranges are detailed. L: liver; B: brain; I: intestine; S: spleen; K: kidneys; GP: gross pathology; HP: histopathology.

Region	Country	Date	Stage	N° Fish GP/ N° Fish HP	Weight (g)	Organs	Main Change	N° Isolates (ID)	Water Temperature (°C)	Cumulative Mortality (%)
CAM	C1	July 2023	FRY	60/06	5.0	Organs pool	Whirling, mortality	C1–A1	32–33 °C	48%
			FRY		5.0			C1–A2		
			PGO	20/07	30.0	I	Intussusception	C1–A3	>32 °C	55%
			PGO		50.0	B, L, K	Hemorrhagic septicemia	C1–A4		
			GO	10/06	600.0	L, K, I	Hemorrhagic septicemia; intussusception	C1–A5		
	C2	April 2023	FRY	60/06	2.0	Organs pool	Whirling, mortality	C2–A1	32–34 °C	52%
			FRY		2.0			C2–A2		
			PGO	20/07	10.0	I	Intussusception	C2–A3	>32 °C	57%
			PGO		30.0	B, L, K	Hemorrhagic septicemia	C2–A4		
			GO	10/06	400.0	B, K, I	Hemorrhagic septicemia; intussusception	C2–A5		
SAM	C3	April 2025	PGO	10/05	150.0	L, S, B	Hemorrhagic septicemia; intussusception	C3–A1	>32 °C	55%
			GO	20/06	180.0			C3–A2		
			GO		300.0			C3–A3		
	C4	November 2023	FRY	60/06	3.0	Organs pool	Whirling, mortality	C4–A1	32–34 °C	50%
			FRY		3.0			C4–A2		
			PGO	20/07	40.0	B, L	Hemorrhagic septicemia; intussusception	C4–A3	>32 °C	47%
			PGO		60.0			C4–A4		
			GO	10/06	500.0		Hemorrhagic septicemia	C4–A5		
	C5	April 2025	PGO	10/05	100.0	B, L, S	Hemorrhagic septicemia	C5–A1	>32 °C	50%
			GO	10/05	400.0			C5–A2		
NAM	C6	August 2021	PGO	20/07	30.0	B, K, I	Hemorrhagic septicemia	C6–A1	>32 °C	54%
			PGO		80.0			C6–A2		
			PGO		90.0			C6–A3		
			GO	20/07	500.0			C6–A4		
			GO		800.0			C6–A5		

**Table 2:** Frequency of clinical and pathological findings in farmed tilapia at different age ranges and production stages in six LAM countries.

Clinical and Pathological Findings	FRY (<5 g; *n* = 180)	PGO/GO (≥5 g; *n* = 180)
Clinical signs		
Loss of appetite or anorexia	75.0%	86.7%
Lethargy and dying individuals on shores	83.9%	79.4%
Erratic swimming behavior, spiraling or uncoordinated movements, loss of buoyancy control	81.7%	87.8%
Abdominal distension	21.7%	68.3%
C-shaped spinal curvature	15.6%	24.4%
Fecal strings (protruded from the anus and/or in varying quantities in the water)	9.4%	25.6%
External inspection		
Head and eyes		
Unilateral or bilateral exophthalmos (pop-eyes)	0.0%	73.9%
Corneal opacity (whitish or opaque eyes)	9.4%	71.7%
Bleeding in the eyes	7.2%	25.6%
Abscesses in the jaw or head region	0.0%	14.4%
Skin and fins		
Hemorrhages at the base of the fins and tail	0.0%	22.8%
Hemorrhagic purulent skin ulcers in the perianal region	0.0%	20.6%
Fin erosion	12.8%	34.3%
Skin darkening	18.9%	66.1%
Petechiae on body surface and operculum	10.6%	24.4%
Gills		
Gill pallor	17.2%	31.7%
Whitish areas on the gill surface	6.1%	37.7%
Internal inspection		
Coelomic cavity		
Serosanguineous ascites	21.1%	51.7%
Multiple abdominal adhesions	0.0%	37.8%
Purulent-appearing material	0.0%	15.6%
Heart		
Pericarditis or whitish discoloration of the heart	0.0%	62.2%
Presence of purulent material in the pericardial sac and/or epicardium	0.0%	67.8%
Brain cavity		
Cerebral edema	18.3%	68.3%
Cerebral hemorrhage	20.6%	66.1%
Presence of yellowish purulent material and meningeal opacity	22.8%	62.8%
Stomach and Intestines		
Hemorrhage and congestion	74.4%	81.1%
Intussusception	43.3%	17.2%
Hepatopancreas		
Irregular appearance and coloration, with pale and congested areas	12.8%	74.4%
Fibrinous adhesions	0.0%	18.3%
Abscesses	0.0%	11.7%
Spleen		
Splenomegaly	9.4%	57.8%
Presence of pale areas	0.0%	9.4%
Kidneys and swim bladder		
Renomegaly	0.0%	7.2%
Pallor	0.0%	63.9%
Gas accumulation in the swim bladder and congestion	18.3%	53.3%
Skeletal muscle		
Abscesses (calcified or not)	0.0%	15.6%

**Table 3:** Description of virulence genes and virulence gene profiles among the 25 SaIa isolates recovered from six LAM countries (arbitrarily named in this study as A, B, and C).

Gene	Product	Main function	C1 (*n* = 5)	C2 (*n* = 5)	C3 (*n* = 3)	C4 (*n* = 5)	C5 (*n* = 2)	C6 (*n* = 5)	Total (*n* = 25)
*spb1*	Spb1 surface protein	Invasion of epithelial cells	+	+	+	+	+	+	100%
*bca*	αC protein (α antigen)	Adherence, invasion, resistance to phagocytosis	+	+	+	+	+	+	100%
*cfb*	CAMP factor	Pore-forming cytolysin	+	+	+	+	+	+	100%
*dltR*	D-alanine regulator	Resistance to antimicrobial peptides	+	+	+	+	+	+	100%
*bac*	βC protein (β antigen)	IgA binding, factor H; immune evasion	+	−	+	+	+	−	60%
*sodA*	Superoxide dismutase A	Protection against oxidative stress	−	−	−	−	−	−	100%
*scpB*	C5a peptidase	Evasion of neutrophil recruitment	−	−	−	−	−	−	100%
Virulence gene profile (*n* = 25)			A	B	A	A	A	B	

Presence (+) and absence (−) of the gene.

**Table 4:** Comparative characterization of ARG detection, antibiotic susceptibility profiles, and MIC values for OTC, FFC, AMX, and ENR in SaIa ST7 CC1 isolates recovered from Nile tilapia in six Latin American countries.

Region	Country	Bacterial ID	ARGs		OTC		FFC		AMX		ENR	
			*tetM*	*tetO*	mm	μm/mL	mm	μm/mL	mm	μm/mL	mm	μm/mL
CAM	C1	C1–A1			S (30)	0.5	S (30)	0.5	S (35)	≤0.5	I (25)	4.0
		C1–A2			S (30)	0.5	S (34)	0.5	S (34)	≤0.5	I (25)	4.0
		C1–A3			S (30)	0.5	S (30)	0.5	S (32)	≤0.5	I (25)	4.0
		C1–A4			S (30)	0.5	S (30)	0.5	S (30)	≤0.5	I (25)	4.0
		C1–A5	+		I (26)	1.0	S (30)	0.5	S (30)	≤0.5	I (24)	4.0
	C2	C2–A1		+	I (22)	4.0	S (32)	1.0	S (35)	≤0.5	I (23)	4.0
		C2–A2		+	I (21)	4.0	S (31)	2.0	S (36)	≤0.5	I (23)	4.0
		C2–A3			I (28)	4.0	S (36)	1.0	S (40)	≤0.5	I (21)	4.0
		C2–A4			I (21)	4.0	S (33)	2.0	S (38)	≤0.5	I (21)	4.0
		C2–A5			I (21)	4.0	S (32)	1.0	S (35)	≤0.5	I (24)	4.0
SAM	C3	C3–A1	+		I (28)	4.0	S (35)	1.0	S (46)	≤0.5	I (22)	4.0
		C3–A2	+		I (28)	4.0	S (36)	<0.5	S (40)	≤0.5	I (21)	4.0
		C3–A3			I (27)	2.0	S (36)	1.0	S (41)	≤0.5	I (22)	4.0
	C4	C4–A1			I (23)	2.0	S (30)	1.0	S (35)	≤0.5	I (21)	4.0
		C4–A2			I (25)	2.0	S (30)	2.0	S (35)	≤0.5	I (21)	4.0
		C4–A3			I (23)	1.0	S (30)	1.0	S (34)	≤0.5	I (23)	4.0
		C4–A4			I (24)	2.0	S (31)	1.0	S (35)	≤0.5	I (21)	4.0
		C4–A5			I (24)	1.0	S (30)	1.0	S (33)	≤0.5	I (21)	4.0
	C5	C5–A1			S (30)	0.5	S (31)	1.0	S (40)	≤0.5	I (25)	4.0
		C5–A2			S (30)	0.5	S (33)	1.0	S (41)	≤0.5	I (23)	4.0
NAM	C6	C6–A1			I (26)	2.0	S (31)	1.0	S (39)	≤0.5	I (26)	4.0
		C6–A2			I (27)	2.0	S (31)	1.0	S (36)	≤0.5	I (24)	4.0
		C6–A3			I (26)	4	S (30)	2.0	S (34)	≤0.5	I (23)	4.0
		C6–A4			I (25)	2	S (31)	1.0	S (35)	≤0.5	I (23)	4.0
		C6–A5			I (24)	2	S (32)	1.0	S (46)	≤0.5	I (24)	4.0

Presence (+) of the gene.

## Data Availability

All MLST sequences obtained were submitted to GenBank and assigned accession numbers PZ024150 to PZ024324, and raw sequencing data are available upon request. All data generated or analyzed during this study are included in this article and its supplementary information files.

## Supplementary Materials

The following supporting information can be downloaded at https://www.mdpi.com/article/10.3390/pathogens15050545/s1, Table S1: Primers used for molecular serotyping, MLST, virulence genes (VGs) and antibiotic resistance genes (ARGs) in 25 strains of *S. agalactiae* Ia isolated between 2021 and 2025 from larvae/fry, juveniles (PGO) and adults (GO) of Nile tilapia that experienced outbreaks of piscine streptococcosis in six Latin American countries.; Table S2: qPCR (CTs) and MLST results for the 25 isolates from the six Latin American countries; Table S3: Systematic comparative analysis of the virulence profiles obtained in this study with the SaIa isolates described in China, Thailand/Vietnam, Indonesia and Egypt; Figure S1: Frequency of microscopic changes observed in different tissues of fish undergoing outbreaks of streptococcosis caused by SaIa ST7 CC1 in all production stages of tilapia farming in six Latin American countries; Figure S2: Results of conventional PCR amplification showing the expected band size for detection of the target virulence gene in the samples analyzed. The amplification products were analyzed by electrophoresis on 1.5% agarose gel with ethidium bromide dye. L1: molecular weight marker; L2–L8: sample amplicons for the respective target gene; C: control. The colored lines show amplicons of the isolates C6-A1 (white), C2-A3 (red), C2-A2 (yellow), and C2-A1 (turquoise).; Figure S3: Results of conventional PCR amplification showing the expected band sizes for the detection of antimicrobial resistance genes in the samples analyzed. The amplification products were analyzed by electrophoresis on 1.5% agarose gel with ethidium bromide dye. L1: molecular weight marker; L2–L10: sample amplicons for the respective target gene; C: control. Amplicons for the tetO gene in isolates C2-A3 (red), C2-A2 (yellow), C6-A1 (white), and C2-A1 (turquoise).
