# Supplementary material for: Streptococcus agalactiae Serotype Ia ST7 CC1 in Farmed Nile Tilapia in Latin America: Age-Dependent Disease Expression and Antimicrobial Susceptibility of an Emerging Clonal Lineage
Source: Pathogens. 2026 May 18;15(5):545. doi: 10.3390/pathogens15050545 (PMC13209410; doi:10.3390/pathogens15050545)

Supplementary Table S1. Primers used for molecular serotyping, MLST, virulence genes (VGs) and antibiotic resistance genes (ARGs) in 25 strains of S. agalactiae Ia isolated between 2021 and 2025 from larvae/fry, juveniles (PGO) and adults (GO) of Nile tilapia that experienced outbreaks of piscine streptococcosis in six Latin American countries.

Gene	Name	Primers	Sequences (5' – 3')	Reference
**Molecular serotyping**				
CpsIa	Capsular polysaccharide	*CpsIa-F*<br>*CpsIa-R*	GGTCAGACTGGATTAATGGTATG<br>TAGCTACTATTCCAGCCCTG	Furfaro et al. (2017)
CpsIb	Capsular polysaccharide	*CpsIb-F*<br>*CpsIb-R*	TATACTTGCCCACCCAAC<br>ACTTATTGGTTTGTGATATTCC	
CpsIII	Capsular polysaccharide	*CpsIII-F*<br>*CpsIII-R*	CGTTATTATGTTACACGCTC<br>CAAGTATGCGATTATCTTCC	
actB	β-Actin	*actB-F*<br>*actB-R*	TGGTGGGTATGGGTCAGAAAG<br>CTGTTGGCTTTGGGGTTCA	Yang et al. (2013)
**Multilocus sequence typing**				
adhP	Alcohol dehydrogenase	adhP-F<br>adhP-R	GTTGGTCATGGTGAAGCACT<br>ACTGTACCTCCAGCACGAAC	Jones et al. (2003
pheS	Phenylalanine-tRNA synthetase	pheS-F<br>pheS-R	GATTAAGGAGTAGTGGCACG<br>TTGAGATCGCCCATTGAAAT	
atr	Amino acid transporter	atr-F<br>atr-R	CGATTCTCTCAGCTTTGTTA<br>AAGAAATCTCTTGTGCGGAT	
glnA	Glutamine synthetase	glnA-F<br>glnA-R	CCGGCTACAGATGAACAATT<br>CTGATAATTGCCATTCCACG	
sdhA	Serine dehydratase	sdhA-F<br>sdhA-R	AGAGCAAGCTAATAGCCAAC<br>ATATCAGCAGCAACAAGTGC	
glcK	Glucose kinase	glcK-F<br>glck-R	CTCGGAGGAACGACCATTAA<br>CTTGTAACAGTATCACCGTT	
tkt	Transketolase	tkt-F<br>tkt-R	CCAGGCTTTGATTTAGTTGA<br>AATAGCTTGTTGGCTTGAAA	
**Virulence-associated genes**				
spb1	Surface protein of GBS	spb1-F<br>spb1-R	GCTGAGACAGGGACAATTAC<br>GTTGAAGGCAACTCAGTACC	Sun et al. (2016)
scpB	C5a peptidase	scpB-F<br>scpB-R	ACAACGGAAGGCGCTACTGTTC<br>ACCTGGTGTTTGACCTGAACTA	
bca	Alpha-subunit protein C	bca-F<br>bca-R	TAACAGTTATGATACTTCACAGAC<br>ACGACTTTCTTCCGTCCACTTAGG	
bac	Beta-subunit protein C	bac-F<br>bac-R	CTATTTTTGATATTGACAATGCAA<br>GTCGTTACTTCCTTGAGATGTAAC	
dltR	Regulatory protein	dltR-F<br>dltR-R	TTGACAGGTCTCTATGATTTAGTC<br>GTCTGGTTCTCAGCCTAATTC	
cfb	Toxins CAMP factor	cfb-F<br>cfb-R	ATCGTTATGGTTTTTACATGA<br>TTATTTTAATGCTGTTTGAAGTG	
sodA	Superoxide dismutase	sodA-F<br>sodA-R	GTAAAACGACGGCCAGT<br>AACAGCTATGACCATG	
**Antimicrobial resistance genes**				
ermB	Erythromycin Ribosomal Methylase	ermB-F<br>ermB-R	GAAAAGGTACTCAACCAAATA<br>AGTAACGGTACTTAAATTGTTTAC	Assane et al. (2025); Mudzana et al. (2021)
ermTR	Erythromycin Ribosomal Methylase	ermTR-F<br>ermTR-R	GAAGTTTAGCTTTCCTAA<br>GCTTCAGCACCTGTCTTAATTGAT	
mefA	Erythromycin resistance efflux pump	mefA-F<br>mefA-R	AGTATCATTAATCACTAGTGC<br>TTCTTCTGGTACTAAAAGTGG	
linB	Lincosamide nucleotidyltransferases	linB-F<br>linB-R	CCTACCTATTGTTTGTGGAA<br>ATAACGTTACTCTCCTATTC	
tetM	Tetracycline resistance	tetM-F<br>tetM-R	GTGGAGTACTACATTTACGAG<br>GAAGCGGATCACTATCTGAG	
tetO	Tetracycline resistance	tetO-F<br>tetO-R	GCGGAACATTGCATTTGAGGG<br>CTCTATGGACAACCCGACAGAAG	
fexA	Phenicol exporter	fexA-F<br>fexA-R	GTACTTGTAGGTGCAATTACGGCTGA<br>CGCATCTGAGTAGGACATAGC(Yang et al., 2013)GTC	
fexB	Phenicol exporter	fexB-F<br>fexb-R	TTCCCACTATTGGTGAAAGGAT<br>GCAATTCCCTTTTATGGACGTT	

Supplementary Table S2. qPCR (CTs) and MLST results for the 25 isolates from the six Latin American countries.

Area/Region	Country	Bacterial ID	qPCR	CT qPCR	*adhP*	*pheS*	*atr*	*glnA*	*sdhA*	*glcK*	*tkt*	MLST
Central America (CAM)	C1	C1-A1	Sala	17,61	10	1	2	1	3	2	2	ST7 CC1
		C1-A2	Sala	17,5	10	1	2	1	3	2	2	ST7 CC1
		C1-A3	Sala	17,23	10	1	2	1	3	2	2	ST7 CC1
		C1-A4	Sala	17,1	10	1	2	1	3	2	2	ST7 CC1
		C1-A5	Sala	17,82	10	1	2	1	3	2	2	ST7 CC1
	C2	C2-A1	Sala	15,99	10	1	2	1	3	2	2	ST7 CC1
		C2-A2	Sala	16,22	10	1	2	1	3	2	2	ST7 CC1
		C2-A3	Sala	15,37	10	1	2	1	3	2	2	ST7 CC1
		C2-A4	Sala	17,17	10	1	2	1	3	2	2	ST7 CC1
		C2-A5	Sala	18,16	10	1	2	1	3	2	2	ST7 CC1
South America (SAM)	C3	C3-A1	Sala	16,51	10	1	2	1	3	2	2	ST7 CC1
		C3-A2	Sala	15,82	10	1	2	1	3	2	2	ST7 CC1
		C3-A3	Sala	16,8	10	1	2	1	3	2	2	ST7 CC1
	C4	C4-A1	Sala	15,17	10	1	2	1	3	2	2	ST7 CC1
		C4-A2	Sala	15,56	10	1	2	1	3	2	2	ST7 CC1
		C4-A3	Sala	15,33	10	1	2	1	3	2	2	ST7 CC1
		C4-A4	Sala	15,39	10	1	2	1	3	2	2	ST7 CC1
		C4-A5	Sala	13,55	10	1	2	1	3	2	2	ST7 CC1
	C5	C5-A1	Sala	18,14	10	1	2	1	3	2	2	ST7 CC1
		C5-A2	Sala	17,16	10	1	2	1	3	2	2	ST7 CC1
North America (NAM)	C6	C6-A1	Sala	19,62	10	1	2	1	3	2	2	ST7 CC1
		C6-A2	Sala	19,21	10	1	2	1	3	2	2	ST7 CC1
		C6-A3	Sala	18,1	10	1	2	1	3	2	2	ST7 CC1
		C6-A4	Sala	18,99	10	1	2	1	3	2	2	ST7 CC1
		C6-A5	Sala	17,6	10	1	2	1	3	2	2	ST7 CC1

Supplementary Table S3. Systematic comparative analysis of the virulence profiles obtained in this study with the SaIa isolates described in China, Thailand/Vietnam, Indonesia and Egypt.

Region / study source	ST / Serotype focus	spb1	bca	cfb (CAMP)	dltR	bac	sodA	scpB	hylB	lmb	cylE	Notes
Latin America (this study)	ST7, serotype Ia	(+) 100%	(+) 100%	(+) 100%	(+) 100%	(+) 60%	−	−	ND	−	ND	Uniform profile across 6 countries; some isolates lacked bac; no scpB or sodA detected.
China (Sun et al. 2016; Su et al. 2019)	ST7, serotype Ia from tilapia	+	+	+	+	+	+	−	ND	−	ND	Most prevalence profile (42.47%): all Ia isolates lacked lmb and scpB but carried cfb and other core factors.
Thailand/Vietnam (Kayansamruaj et al. 2015; Legario et al. 2020)	ST7, serotype Ia from tilapia	ND	+	+	ND	ND	+	−	+	+/−	+	Thai ST7 share a conserved virulence island; hylB and cylE frequently present; scpB generally absent
Indonesia (Sukenda et al. 2021; Syuhada et al. 2020)	Mixed biotypes; Ia ST7 present	ND	+	+	ND	ND	ND	variable	+ (β-hemolytic strains)	+	+	β-hemolytic ST7 strains carried more virulence genes (cylE, hylB, lmb, fbsA/B, cfb) than non-hemolytic isolates
Egypt (Algammal et al. 2025)	Mixed STs; Ia ST7 included	ND	ND	(+) 100%	ND	ND	ND	ND	(+) 82.1%	(+) 78.5%	(+) 57.1%	High prevalence of cfb, hylB, lmb, cylE among tilapia isolates; study includes ST7 but also other lineages

Key: “ND” = not detailed/consistent in the cited source for ST7 specifically; “+” = commonly present; “−” = commonly absent.

Supplementary Figure S1. Frequency of microscopic changes observed in different tissues of fish undergoing outbreaks of streptococcosis caused by SaIa ST7 CC1 in all productive stages of tilapia farming in six Latin American countries.

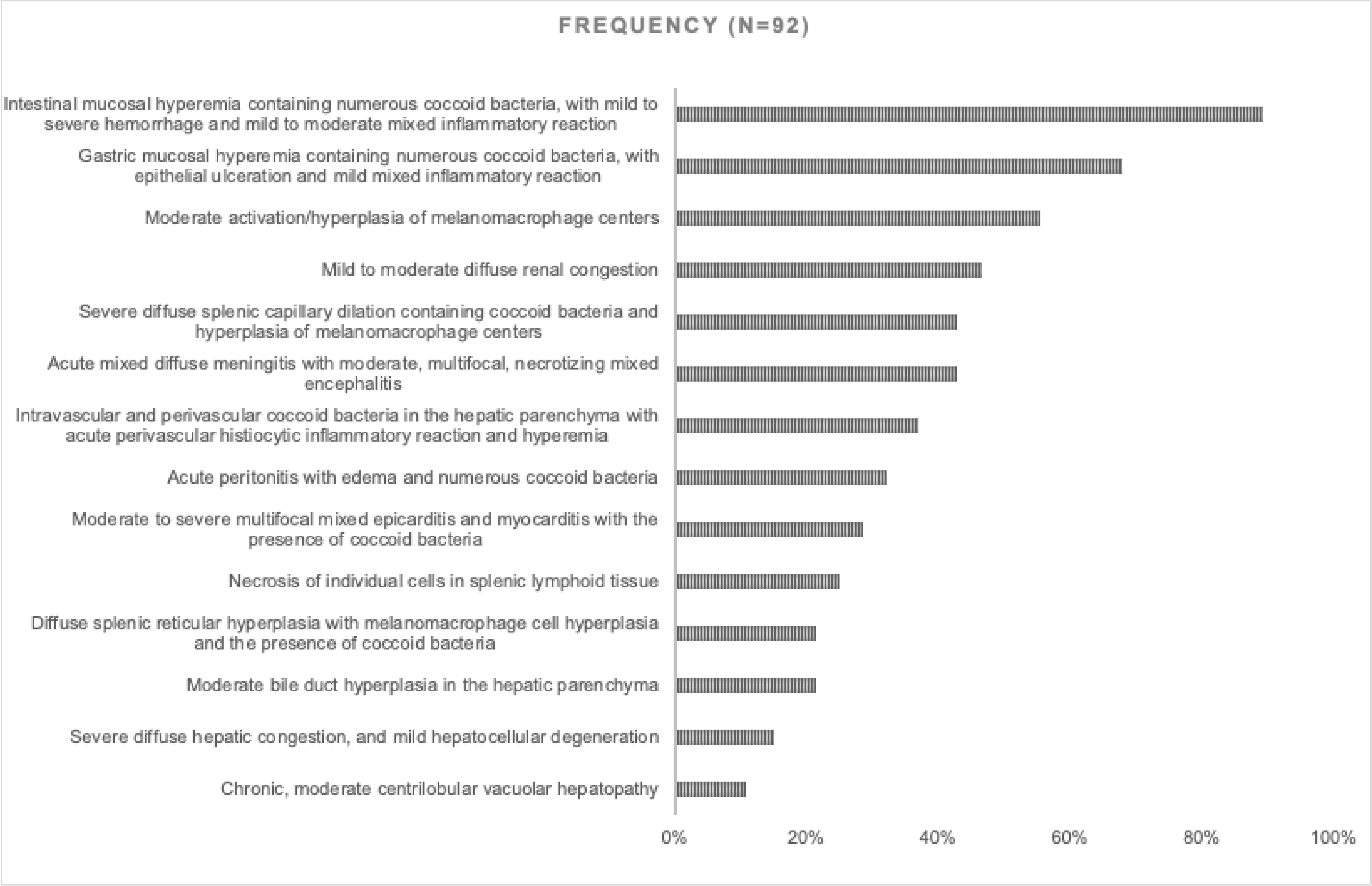

Supplementary Figure S2. Results of conventional PCR amplification showing the expected band size for detection of the target virulence gene in the samples analyzed. The amplification products were analyzed by electrophoresis on 1.5% agarose gel with ethidium bromide dye. L1: molecular weight marker; L2-L8: sample amplicons for the respective target gene; C: control. The colored lines show amplicons of the isolates C6-A1 (white), C2-A3 (red), C2-A2 (yellow), and C2-A1 (turquoise).

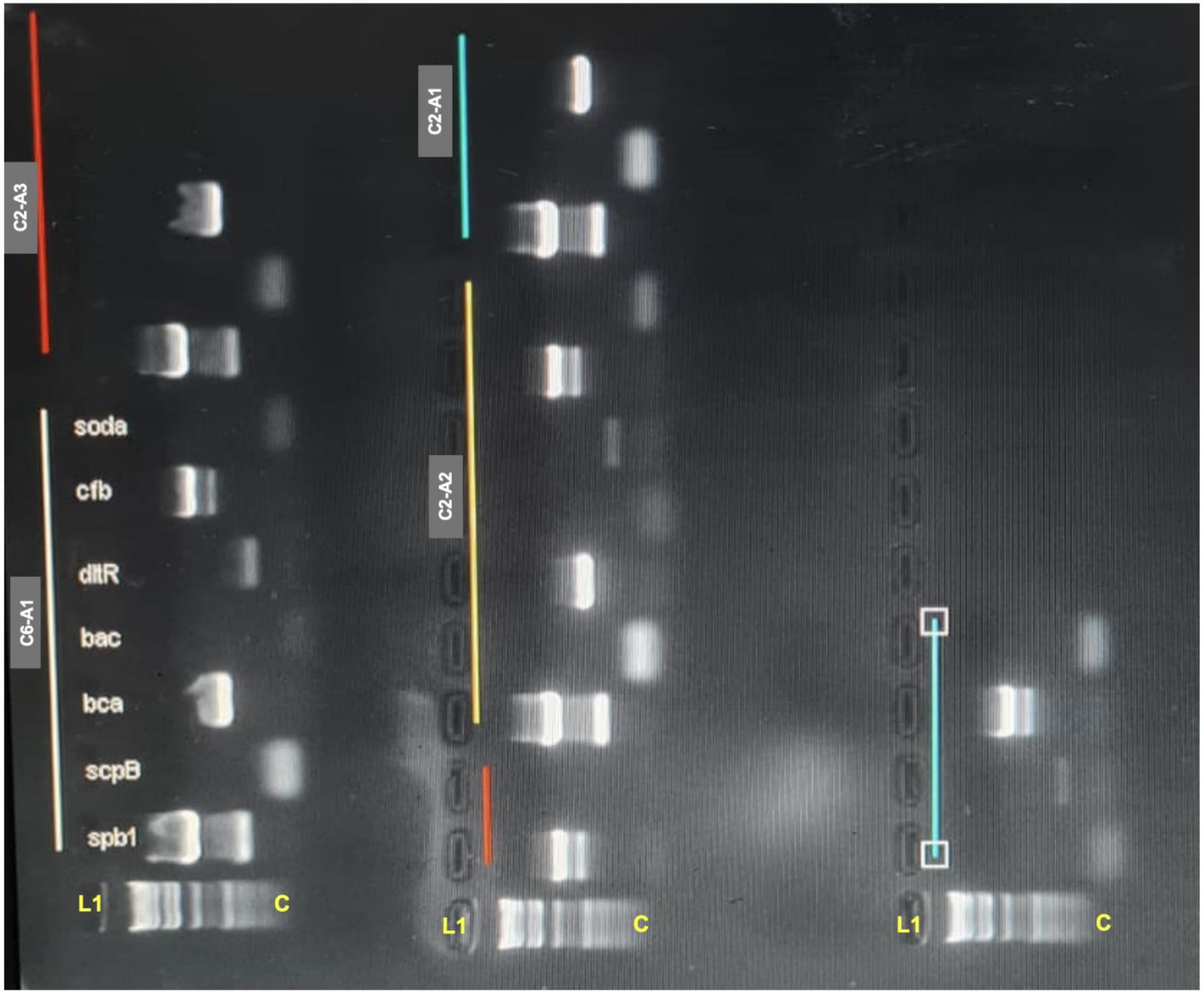

Supplementary Figure S3. Results of conventional PCR amplification showing the expected band sizes for the detection of antimicrobial resistance genes in the samples analyzed. The amplification products were analyzed by electrophoresis on 1.5% agarose gel with ethidium bromide dye. L1: molecular weight marker; L2-L10: sample amplicons for the respective target gene; C: control. Amplicons for the tetO gene in isolates C2-A3 (red), C2-A2 (yellow), C6-A1 (white), and C2-A1 (turquoise).

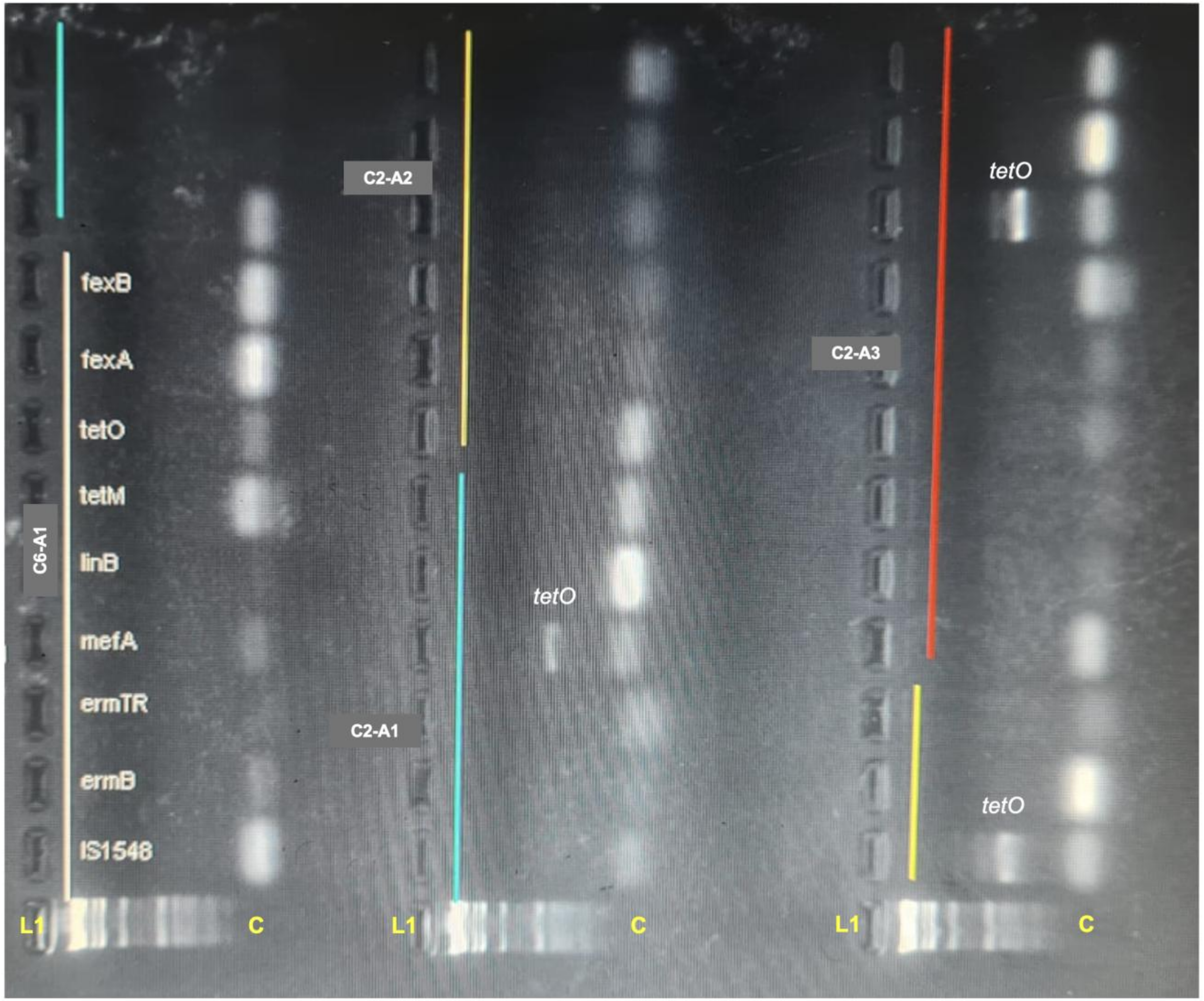

Supplement: Supplementary file 1 [file pathogens-15-00545-s001.zip › pathogens-4294285-supplementary.pdf]
